# Seroprevalence of human T-lymphotropic virus infection among blood donors in China: a first nationwide survey

**DOI:** 10.1186/s12977-020-00546-w

**Published:** 2021-01-07

**Authors:** Le Chang, Shanhai Ou, Zhengang Shan, Faming Zhu, Huimin Ji, Xia Rong, Fei Guo, Xinyi Jiang, Huizhen Sun, Ying Yan, Lunan Wang

**Affiliations:** 1 National Center for Clinical Laboratories, Beijing Hospital, National Center of Gerontology; Institute of Geriatric Medicine, Chinese Academy of Medical Sciences, Beijing, People’s Republic of China; 2 Beijing Engineering Research Center of Laboratory Medicine, Beijing Hospital, Beijing, People’s Republic of China; 3 Xiamen Blood Center, Xiamen, People’s Republic of China; 4 Guangzhou Blood Center, Guangzhou, People’s Republic of China; 5 Transfusion Research institute, Blood Center of Zhejiang Province, Hangzhou, People’s Republic of China; 6 Graduate School, Peking Union Medical College, Chinese Academy of Medical Sciences, Beijing, People’s Republic of China

**Keywords:** Human T-lymphotropic virus, Blood donor, Seroprevalence

## Abstract

**Background:**

So far, the prevalence of human T-lymphotropic virus (HTLV) type 1 and 2 in some highly populated countries such as China is still unknown. In this study, a multi-center nationwide serological survey was designed and performed, to reveal the seroprevalence of HTLV infection among Chinese blood donors.

**Results:**

Among 8,411,469 blood donors from 155 blood establishments, 435 were finally confirmed as HTLV carriers. The prevalence of HTLV infection in China varied in different provinces: Fujian had the highest prevalence of 36.240/100,000 (95% CI 31.990–41.050) and eleven provinces did not find HTLV-seropositive donors in the three years. no HTLV-2 infection was found. The overall prevalence of HTLV-1 in China decreased from 2016 to 2018. Female was identified as an independent risk factor of HTLV infection in China. Besides, seroconversion was observed in two of seven seroindeterminate donors 85 and 250 days after their last donation, respectively.

**Conclusions:**

The seroprevalence of HTLV infection in most areas of China among blood donors is quite low, but it varies significantly in different geographic areas. Screening anti-HTLV-1/2 antibody and follow-up of serointederminate donors are essential to ensure blood safety especially in areas where we have found HTLV infected donors.

## Background

Human T-lymphotropic virus type 1 (HTLV-1) was the first found human retrovirus in the early 1980s by Gallo et al. [1]. Although most HTLV-1 infected cases are asymptomatic, previous studies pointed that 5–10% of infections suffered from adult T-cell leukemia lymphoma (ATL) [2] and HTLV-1 associated myelopathy or tropical spastic paraparesis (HAM/TSP) [3], which reducing patients’ life quality and even threatening public health.

HTLV-1 shows a unique endemic distribution in the world, with high endemic rates of infection in southwestern Japan, the Caribbean islands, Central and South America, Central Australia, sub-Saharan Africa, Romania and parts of the Middle East. It is estimated that there are at least 5–10 million HTLV-1 infected individuals [4]. However, data from some highly populated countries and regions such as China were not available. Thus, the current total number of HTLV-1 carrier is probably underestimated [5, 6].

Since HTLV was found as a member of the transfusion-transmitted infectious pathogen [7], blood donor screening for anti-HTLV antibody was first introduced in Japan in 1986. Two years later commercial HTLV-1 antibody assay became available in the United States [8], then followed by other countries. So far, many studies on the prevalence of HTLV infection among blood donors worldwide were achieved. It was reported that Japan had the highest seroprevalence of HTLV-1 infection with 6.6/1000 in men and 10.2/1000 in women [9]. Others such as the United States, UK, and France with relatively low prevalence in first-time blood donors, 5.1/100,000, 4.7/100,000, and 4.8/100,000, respectively [10, 11].

In China, previous regional studies showed that a prevalence ranged from 16.9/100,000 to 99/100,000 in Fujian province [12–16]. Whereas the prevalence of other regions in China is still unknown due to the lack of multicenter large-scale surveys. Therefore, it is difficult to estimate the overall burden of HTLV infection to China, which may cause a probably underestimated total number of HTLV-1 carrier worldwide [5, 6].

HTLV-1/2 antibody assay has been introduced in certain blood establishments in China since 2006. In Fujian, Guangdong, and Zhejiang province, which were estimated to have a high prevalence of HTLV-1 infection, all blood donors were tested HTLV-1/2 antibody routinely. While, in other provinces, no less than 10% of donation samples were randomly selected and screened every year. In this study, we reported the national epidemiological data from 8.4 million blood donors screened by HTLV-1/2 antibody among 155 blood establishments from 29 provinces or municipalities from 2016 to 2018.

## Results

### Screening and confirmatory results

From 2016 to 2018, 8,411,469 blood donors were routinely screened anti-HTLV-1/2 antibodies all over China. In Fujian, Guangdong (except Shenzhen), and Zhejiang province, 4,497,616 blood donors were tested, among which 2629 were repeatedly reactive by screening tests and 370 donors were finally confirmed as HTLV-1 infection by a confirmatory test.

**Fig. 1:**
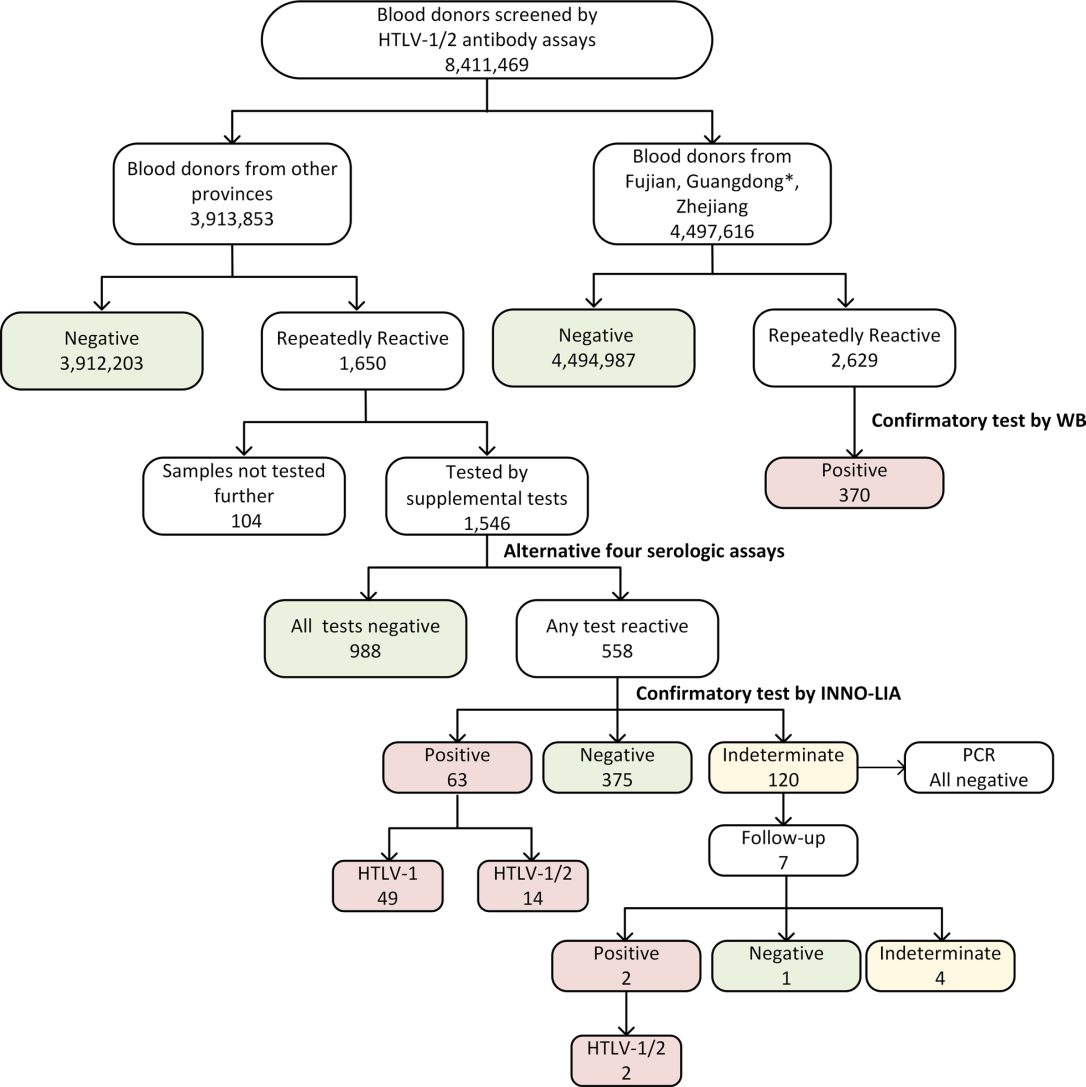
Screening and confirmation algorithm of HTLV infection. *Since samples from Shenzhen were confirmed in National Center for Clinical Laboratories (NCCL), data of Shenzhen was not included in that of Guangdong province

In other areas in China, 3,913,853 blood donors were tested and 1650 donors were repeatedly reactive. 1546 samples were sent to the National Center for Clinical Laboratories (NCCL) for further testing and 104 samples weren’t tested because of lack of volume or other reasons. 63.9% (988/1546) samples were non-reactive by all four different supplementary assays, while 558 samples were reactive by at least one assay and then tested by line immunoassay. 63 samples were positive by the confirmatory test, among which 49 donors were discriminated as HTLV-1 infection and 14 were untypeable HTLV infection (Additional file 1: Table S1). 120 samples were seroindeterminate but all were PCR-negative. Seven of these initial indeterminate donors were followed at least 8 weeks after the last donation. Two of them were confirmed positive (both were HTLV-1/2), and one was negative. However, the results from the other four donors were still indeterminate. All the screening and confirmation algorithm was shown in Fig. 1.

### Prevalence of HTLV-1/2 infection in China

**Fig. 2:**
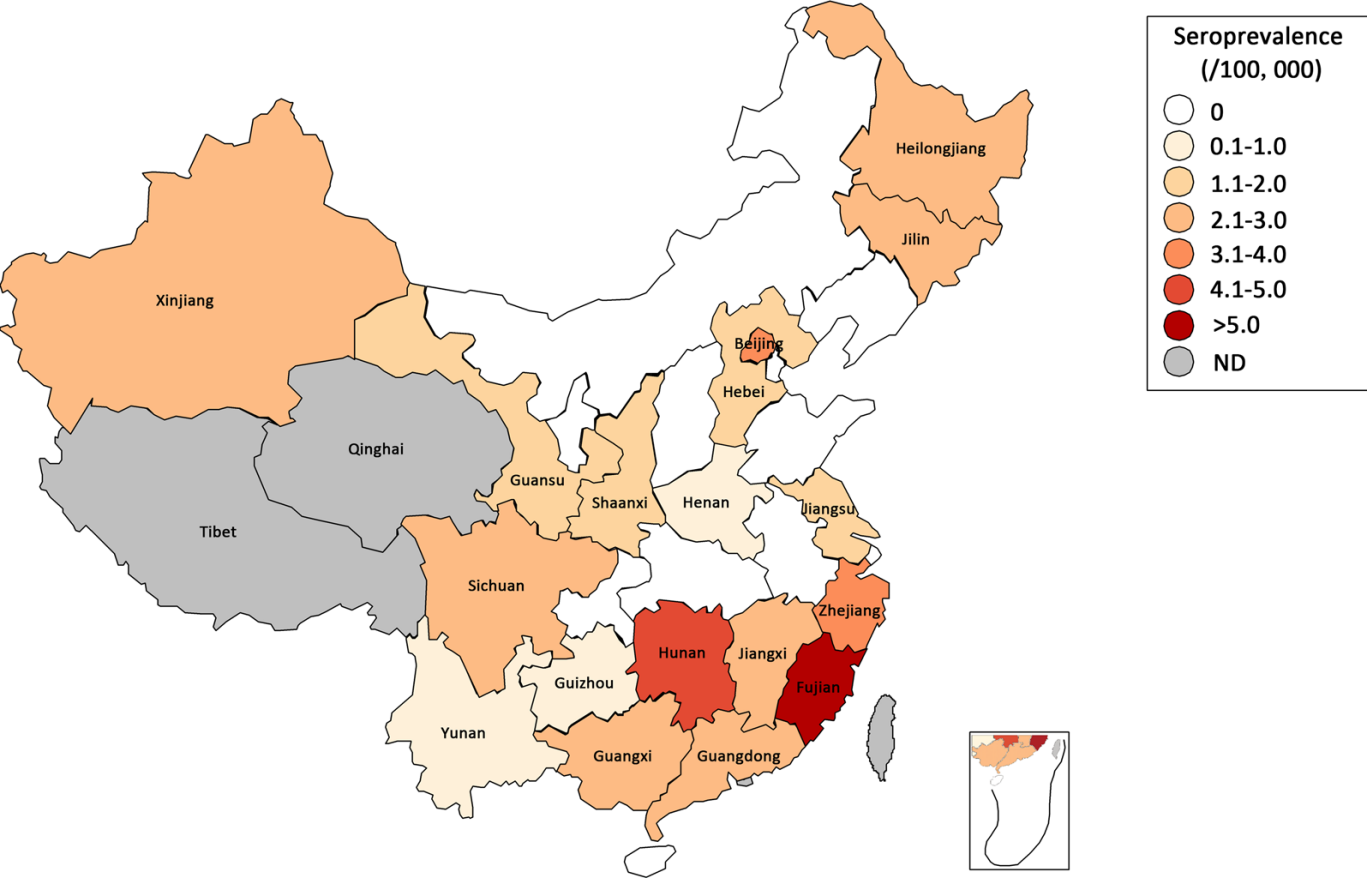
Distribution of HTLV seroprevalence among blood donors all over China. Different color represented different seroprevalence of HTLV infection. Two provinces in grey, Qinghai and Tibet, didn’t screen anti-HTLV-1/2 antibody during the 3 years

Finally, 435 donors were confirmed as HTLV-1/2 carriers. Fujian province owned the highest prevalence of 36.240/100,000 (247/681,564, 95% CI 31.990–41.050). While the prevalence of all other areas in China was only 2.432/100,000 (188/7,729,905, 95% CI 2.108–2.805), nearly one-fifteenth of that of Fujian (Table 1; Fig. 2). Followed that of Fujian, the HTLV prevalence in Hunan province, Zhejiang province, and Beijing were more than 3/100,000, a bit higher than in other areas. Noteworthily, the prevalence in Guangdong was only 2.867/100,000 (95% CI 2.301–3.573), while the prevalence in Shenzhen, a large city located in Guangdong, was significantly higher (3.713/100,000, 95% CI 2.124–6.490), similar to that of Beijing. HTLV infections were not found in eleven provinces in China during the 3 years.

**Table 1:** Blood donations screened by HTLV-1/2 antibody in China

Province/City	Number of screened blood donors^a^	Number of repeated reactive by ELISA	Number of donations not tested further	Supplemental test					Seroprevalence (/100,000)	95%CI (/100,000)
				Negative	IND	Positive				
						HTLV-1	HTLV-1/2	Total		
Fujian	681,564 (2.53%)	939	0	692	–	247	–	247	36.240	31.990–41.050
Hunan	164,090 (0.37%)	99	6	74	11	7	1	8	4.875	2.471–9.621
Zhejiang	1,384,135 (3.49%)	460	0	404	–	56	–	56	4.046	3.116–5.253
Beijing	124,326 (0.80%)	23	1	16	2	4	0	4	3.217	1.251–8.273
Jiangxi	240,310 (0.81%)	107	0	91	9	5	2	7	2.913	1.411–6.013
Guangxi	171,951 (0.57%)	112	1	100	6	4	1	5	2.908	1.242–6.807
Guangdong	2,755,108 (3.60%)	1322	5	1231	7	78	1	79	2.867	2.301–3.573
Sichuan	116,388 (0.22%)	83	0	73	7	2	1	3	2.578	0.877–7.578
Xinjiang	38,965 (0.26%)	35	0	32	2	1	0	1	2.566	0.453–14.540
Jilin	182,328 (0.89%)	72	24	39	5	3	1	4	2.194	0.853–5.641
Heilongjiang	182,739 (0.64%)	35	0	27	4	2	2	4	2.189	0.851–5.628
Hebei	202,170 (0.40%)	140	12	120	4	2	2	4	1.979	0.770–5.087
Gansu	56,325 (0.32%)	16	5	7	3	1	0	1	1.775	0.313–10.060
Jiangsu	294,997 (0.53%)	63	0	50	9	2	2	4	1.356	0.527–3.487
Shaanxi	82,711 (0.31%)	27	0	26	0	1	0	1	1.209	0.213–6.848
Guizhou	413,072 (1.92%)	204	2	188	10	3	1	4	0.968	0.377–2.490
Yunnan	128,279 (0.41%)	100	19	78	2	0	1	1	0.780	0.138–4.416
Henan	267,149 (0.43%)	111	4	95	10	1	1	2	0.749	0.205–2.730
Anhui	142,604 (0.36%)	68	3	62	3	0	0	0	0	0.000-2.693
Hainan	35,564 (0.60%)	17	0	17	0	0	0	0	0	0.000-10.800
Hubei	138,704 (0.34%)	38	2	32	4	0	0	0	0	0.000-2.769
Liaoning	98,876 (0.31%)	26	1	24	1	0	0	0	0	0.000-3.885
Innermengolia	21,062 (0.11%)	20	8	10	2	0	0	0	0	0.000-18.230
Ningxia	30,490 (0.70%)	8	0	4	4	0	0	0	0	0.000-12.600
Shandong	251,019 (0.38%)	94	9	79	6	0	0	0	0	0.000-1.530
Shanxi	55,651 (0.22%)	24	0	22	2	0	0	0	0	0.000-6.901
Shanghai	69,479 (0.40%)	11	0	10	1	0	0	0	0	0.000-5.528
Tianjin	51,394 (0.51%)	19	2	14	3	0	0	0	0	0.000-7.473
Chongqing	30,019 (0.16%)	6	0	6	0	0	0	0	0	0.000-12.790
Total	8,411,469	4279	104	3623	117	419	16	435	5.172	4.708–5.687

*IND* indeterminate

^a^The proportion of individuals tested anti-HTLV antibody among all 18–60 year-old adults of the general population based on the data from 6th China nationwide population census of each province in 2010

**Fig. 3:**
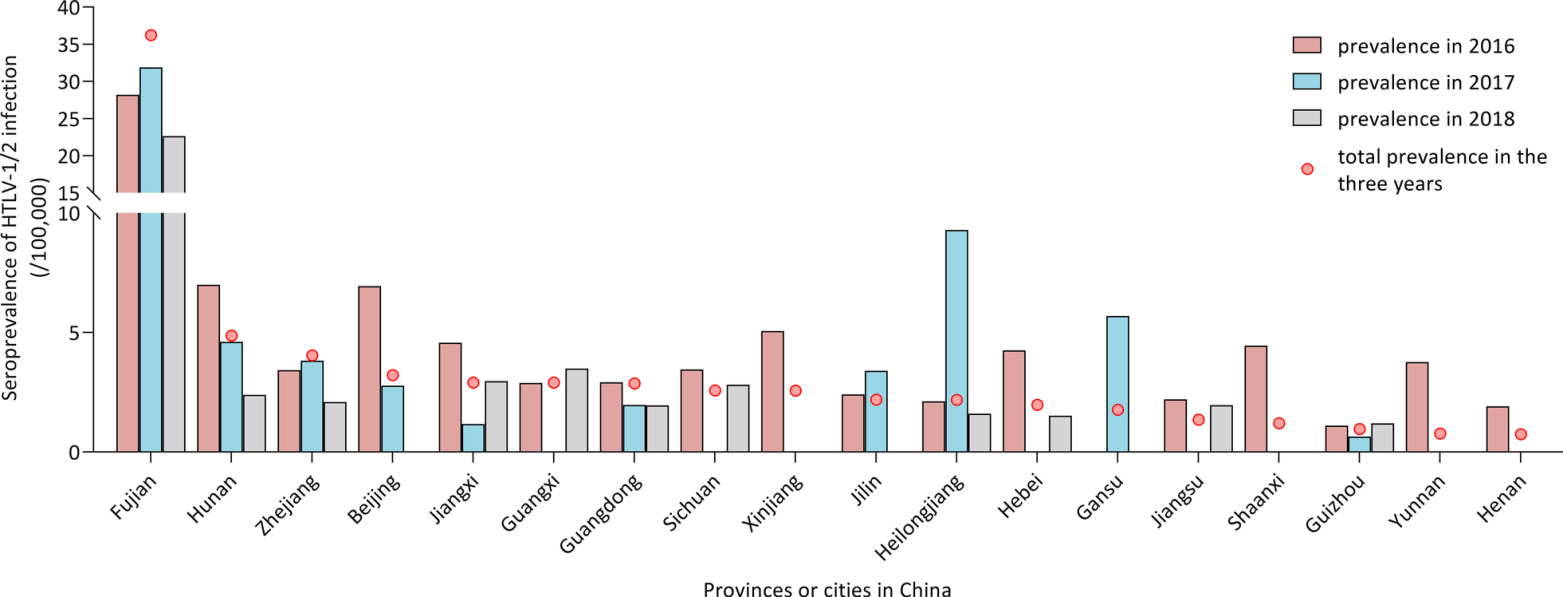
The fluctuating seroprevalence of HTLV infection among blood donors in 2016 to 2018. All the data from provinces or cities with a confirmed HTLV-1/2 infection in the 3 years were shown. The nationwide prevalence of anti-HTLV-1/2 antibody gradually reduced from 5.016/100,000 in 2016 and 4.951/100,000 in 2017 to 3.469/100,000 in 2018

From 2016 to 2018, fluctuating seroprevalence was shown in Fig. 3. The total prevalence of HTLV-1/2 antibody gradually reduced from 5.016/100,000 (95% CI 4.303–5.847) in 2016 to 4.951/100,000 (95% CI 4.226–5.800) in 2017 then to 3.469/100,000 (95% CI 2.899–4.151) in 2018. However in Fujian province, the seroprevalence of HTLV infection first suffered a slight increase from 28.205/100,000 (95% CI 22.700-35.050) in 2016 to 31.883/100,000 (95% CI 26.140–38.890) in 2017, and then declined to 22.646/100,000 (95% CI 17.900-28.660) in 2018.

Besides, we found the positive prediction value (PPV) of the screening HTLV-1/2 antibody assays varied from 0–26.30% in different provinces, with an average value of 10.17% over all samples. Among 63 confirmed positive samples in NCCL, the positive detection rates of the four screening assays were 71.43% (R1), 84.13% (R2), 90.00% (R3), and 88.46% (R4), respectively.

### Risk factors of HTLV infection

Demographic Information (age, sex, donation history, and ethnicity) from 416 to 435 confirmed HTLV-1/2 infection donors were acquired and analyzed. Compared with all blood donors’ distribution [17], HTLV-1/2 infection was independently associated with female in China (Table 2). Women have a 1.307-fold (95% CI 1.076–1.587) risk than men donors to be infected by HTLV-1/2. And compared with repeat donors, first donors seemed more likely to be anti-HTLV antibodies positive (odd ratios, OR: 1.779, 95% CI 1.439–2.199). However, we analyzed the seroprevalence of confirmed positive first donors and repeat donors among different blood establishments, and found that the prevalence of first donors was 3-fold higher than that of repeat donors in Fujian province, while in other blood establishments no difference was observed (2.223/100,000 in first donors vs. 2.160/100,000 in repeat donors), which may result from the fact that confirmed positive donors could not donate blood again. Age and ethnicities showed no risk effect on HTLV-1/2 infection.

**Table 2:** Demographic characteristics of HTLV seropositive blood donors

Characteristics	HTLV-1/2 positive donors (n = 416, seroprevalence/100,000)	OR (95% CI)	p value
Age, year			0.091
18–25	123 (5.356)	1.000	
25–35	117 (5.329)	0.995 (0.772–1.282)	0.969
36–45	110 (5.169)	0.965 (0.746–1.248)	0.786
46–55	61 (3.590)	0.670 (0.493–0.911)	0.010
> 55	5 (5.404)	1.009 (0.413–2.647)	0.985
Sex			
Male	238 (4.449)	1.000	
Female	178 (5.814)	1.307 (1.076–1.587)	0.007
Previous donation history			
Repeat donors	120 (3.405)	1.000	
First-time donors	296 (6.057)	1.779 (1.439–2.199)	< 0.0001
Ethnicity			
Han	398 (4.939)	1.000	
Non-Han	18 (5.095)	1.032 (0.643–1.654)	0.897

### Follow-up of indeterminant and confirmed positive donors

Seven from 120 initial indeterminate donors by INNO-LIA were followed once from 85 days to 302 days after their last donation (Table 3). All the seven donors confirmed as indeterminate due to the only one band of gp21 I/II antigen at the first confirmation test. Two donors made a seroconversion with the appearance of p19 I/II antigen 85 days and 250 days after their last donation, however, we did not find HTLV proviral DNA from these two whole blood samples. The signal to cutoff value (S/CO) or cutoff index (COI) values of 4 screening tests between the twice results increased obviously. While changes of S/CO or COI values from the other five donors were inconspicuous and the confirmation result from Donor 1442 changed to negative. Four initial indeterminate donors were still indeterminate after the first follow-up.

**Table 3:** Follow-up of initial indeterminate donors (n = 7) by confirmatory test

No.	Province	Sex	Age	Follow-up (day)	Supplemental tests (R/NR, S/CO or COI)				INNO-LIA							PCR result	Result
									Confirmation				Discrimination				
					R1	R2	R3	R4	p19 I/II	p24 I/II	gp46 I/II	gp21 I/II	p19-I	gp46-I	gp46-II		
177	Jiangsu	F	36	0	NR 0.43	NR 0.76	R 3.71	R 6.10	–	–	–	1+	–	–	–	UD	IND
				85	NR 0.48	NR 0.54	R 4.11	R 9.50	1+	–	––	±	–	–	–	UD	HTLV-1/2
1342	Heilongjiang	M	52	0	NR 0.65	R 4.89	R 171.50	R 19.40	–	–	–	2+	–	–	–	UD	IND
				250	R 1.11	R 5.51	R 142.10	N/A	±	–	–	2+	–	–	–	UD	HTLV-1/2
91	Heilongjiang	M	50	0	NR 0.15	NR 0.56	R 42.37	NR 0.50	–	–	–	1+	–	–	–	UD	IND
				137	NR 0.17	NR 0.90	R 59.43	NR 0.40	–	–	–	±	–	–	–	UD	IND
513	Sichuan	M	37	0	NR 0.25	R 1.13	R 34.48	R 50.00	–	–	–	2+	–	–	–	UD	IND
				151	NR 0.23	R 1.04	R 66.61	R 49.10	–	–	–	±	–	–	–	UD	IND
1357	Henan	M	46	0	NR 0.17	NR 0.68	R 197.10	NR 0.20	–	–	–	±	–	–	–	UD	IND
				126	NR 0.12	NR 0.38	R 213.20	N/A	–	–	–	±	–	–	–	UD	IND
1439	Jiangsu	M	19	0	NR 0.21	R 2.05	R 27.30	R 1.70	–	–	–	±	–	–	–	UD	IND
				302	NR 0.13	NR 0.35	NR 0.20	R 1.30	–	–	–	±	–	–	–	UD	IND
1442	Jiangsu	F	31	0	NR 0.35	R 2.85	NR 0.61	R 1.10	–	–	–	±	–	–	–	UD	IND
				203	NR 0.19	R 2.91	NR 0.60	NR 0.20	–	–	–	–	–	–	–	UD	Negative

*R* reactive, S/CO or COI ≥ 1.0; *NR* nonreactive, S/CO or COI < 1.0;* UD* undetected;* IND* indeterminate;* N/A* not available;* F* Female;* M* Male

All the HTLV-1/2 carriers tested by NCCL were contacted to complete a more targeted questionnaire after validation of HTLV infection. Finally, we got 13 questionnaires back. 10 of 13 donors were local people, and another three were born in Heilongjiang (donated blood in Jilin), Fujian (donated blood in Shenzhen), and Hunan (donated blood in Shenzhen), respectively. 12 of 13 donors denied having been to any HTLV-endemic countries, except one who had a short visiting history in Japan. Four of 13 HTLV carriers have been to Fujian: one stayed there for three years, another had annual short visiting, and the information of the other two donors was unknown. 11 of 13 donors have experienced invasive procedures, such as surgery, transfusion, acupuncture, or tooth washing. No epidemiological association was found in those HTLV-1/2 carriers through targeted questionnaires.

## Discussion

HTLV-1 infection is still a neglected disease [18], and also has not been fully recognized in China until 2016. This study was the first nationwide multicenter large-scale survey on the seroprevalence of HTLV infection among Chinese blood donors. Since not all samples in all blood establishments in China were enrolled, there may be a statistical sampling bias in the study. Although we could not calculate the exact seroprevalence of HTLV infection among blood donors all over China, while according to the numbers of positive donors in different areas in the study, the seroprevalence of most provinces was lower than HTLV-1 prevalence in reported low-endemic areas such as the United States, UK, and France [10, 11]. In Asia, Japan and Iran are among the high-endemic countries [6, 19], and other regions are medium- or non-endemic of HTLV. Our study suggested that most areas in mainland China belong to the non-endemic area, with a similar prevalence to Korea (7/100,000) [20] and Hong Kong China (4.1/100,000) [21].

In the study, 14 confirmed positive donations couldn’t be discriminated into HTLV-1 or HTLV-2. Since all other HTLV positive donors were HTLV-1 carriers, therefore, we presumed that these untypeable HTLV infections were probably HTLV-1 infection, and their low titer of HTLV antibodies led to untypeable results. According to previous studies in China, only three HTLV-2 carriers were found in 5480 samples, among which two HTLV-2 infections were from the high-risk group and one were from 3548 blood donors in two blood centers from Henan and Hubei [22], however, no further information was provided from the study including WB strip picture or HTLV-2 genome sequences from the three samples. Therefore, in China, HTLV-1 is the main type of HTLV infection among blood donors.

The HTLV prevalence all over China varies significantly in different geographic areas. In 2019, Li et al. [23] reported the seroprevalence of HTLV-1/2 infection (2.51/100,000, 95% CI 1.46–3.56) among 875,453 blood donors from 20 blood centers in China and showed a quite different prevalence in different regions of China. Another cross-sectional study in 2005 reported a total of 19 confirmed HTLV-1 positives among 145,293 blood donors from 13 provinces in China and found that all these HTLV-1 carriers were from Fujian province [12]. In our study we also found that Fujian had a quite higher prevalence of 36.240/100,000 (95% CI 31.990–41.050) compared with other areas of mainland China, however, it was still much lower than those in Japan, the Caribbean area, or Africa [5] but similar to that of Taiwan [24]. In 2015, Xie et al. [15] screened 253,855 blood donations in Fujian from 2004 to 2013 and found 43 HTLV-1 infections with a seroprevalence of 16.9/100,000, which was similar to our study. Except for Fujian, some regions geographically adjacent to Fujian, such as Hunan, Zhejiang, Jiangxi, and Guangxi, and some large cities, such as Shenzhen and Beijing, also have relatively high HTLV prevalence, which might cause by population mobility. Genetic analysis of HTLV-1 found in Fujian [15] revealed that most of the isolates were transcontinental subtype of genotype A and they shared the same but unique gp46 L55P mutation with isolates from Japan and Taiwan, which was not presented in other regions over the world, thus indicating the origin of HTLV-1 in Fujian may be introduced from Japan or Taiwan or they shared the same ancestor.

Besides, we found that PPVs of the repeatedly reactive samples were higher in regions with higher seroprevalence of HTLV infection and different screening assays showed different performance on samples collected from non-epidemic areas. Two assays based on the ELISA method (R1 and R2) showed unsatisfied positive detection rates using confirmed positive samples. Furthermore, our previous study showed remarkable different PPVs of these four assays, varying from 21.0% (R3) to 91.7% (R1) [25]. Therefore, a special strategy should be applied to screen samples from non-epidemic areas to avoid both false negatives and false positives results.

From this study, compared with information from all blood donor population [17], limited demographic data of HTLV positive donors indicated that female was an independent risk factor of HTLV infection. Many studies suggested that women usually had a higher HTLV prevalence than men for the special male-to-female transmission route of HTLV: In Japan, the overall women-to-men ratio of the HTLV-1 incidence density was 3.0 [26]; the prevalence of HTLV in West Africa was 1.5% (16/1,089) for men and 3.7% (55/1,494) for women, and female sex was a strong risk factor of HTLV positivity [27]. More first donors were confirmed HTLV-1/2 infection in the study. Noteworthily, almost all blood donors including repeat donors in China were not tested anti-HTLV-1/2 antibodies except those from Fujian province, before this large-scale investigation. We found that in Fujian the prevalence of first donors was threefold higher than that of repeat donors, while the phenomenon was not observed in other provinces. Since a few studies on the prevalence of HTLV conducted in Fujian before 2016, all identified positive donors before or during the study could not donate blood anymore, which may explain the significantly higher positive rate among first-time donors than among repeat donors. The gradually decreasing data from 2016 to 2018 also indicated that with more and more HTLV-1/2 infected donors found, the majority of repeat donors were seronegative and that the first donors are not a real risk for HTLV infection.

A high proportion of indeterminate results of HTLV confirmation has always been a challenge worldwide. In our study, among 558 donations further tested by INNO-LIA, 120 were seroindeterminate. Previous studies reported different rates of seroconversion of indeterminate donations. Martins et al. [28] made a 4-year follow-up of 60 blood donors with a WB-indeterminate result and found that 12 cases (12/60, 20%) finally showed seroconversion and 48 donations were false-positive by the first test. In HTLV-endemic areas, such as Zaire or Central Africa, the true positive rate of HTLV-indeterminate donors was as high as 68% and 65.56%, respectively [29, 30]. However, in non-endemic areas the positive rate decreased to 8.2% according to a Japan study [31]. Therefore, the PCR method was involved in identifying the true status of one with indeterminate HTLV results by a confirmatory test. Whereas, Busch et al. [32] found that only six (1.4%) of 425 HTLV indeterminate specimens could be confirmed by PCR, which is similar to our results that we found no PCR-positives among all the indeterminate samples. In the present study, through follow-up, we finally found two seroindeterminate donors realized seroconversion 85 days and 250 days after their last donations. Four cases remained an indeterminate result after 126–302 days and we recommended these donors to be deferred temporally and continue to be followed due to the evidence that low proviral load and mutations of HTLV-1 genome may lead to a seroindeterminate result of confirmatory test and this situation may last for a long time [31, 33]. Besides, false indeterminate results should not be ignored. Therefore, a follow-up strategy is of great necessity in low-epidemic areas.

There were several limitations in the study. First of all, due to the large population of donors in China, we could not use a consistency algorithm in the HTLV antibody testing across all of the blood establishments. Therefore, in the study three initial screening assays and two different confirmatory assays were involved, which may influence the true seroprevalence of HTLV-1/2 infection in different regions of China. Secondly, the seroprevalence of our study was derived from data among healthy blood donors, which is a specific population that may have different demographic characteristics to the general population. And due to the limitation of the study population, the seroprevalence of children, teenagers, or the old people (aged > = 60 years old) were unable to be estimated from this study. In the third, only a few donors with indeterminate results and confirmed donors were successfully followed. The true infection status of the others was unknown. In addition, since this study was based on the investigation of seroprevalence of HTLV-1/2 infection, not all the initial positive samples were tested HTLV-1/2 proviral DNA by PCR method, and we did not get the HTLV sequence of the confirmed positive samples either. Therefore, we couldn’t analyze the origin of the virus among blood donors in China.

## Conclusions

In summary, the HTLV prevalence of most areas among blood donors in China is as low as other non-endemic countries or areas. The prevalence varies significantly in different geographic areas all over China and Fujian has a higher prevalence compared with other areas. Screening anti-HTLV-1/2 antibody and follow-up of serointederminate donors are essential to ensure blood safety, especially in areas where we have found HTLV infected donors. Strategy on screening and confirmation of HTLV infection in Chinese blood donors should be made and applied especially in some provinces with high prevalence.

## Methods

### Study population

From 2016 to 2018, blood donations from 155 blood establishments all over mainland China except Qinghai and Tibet screened HTLV-1/2 antibody after routinely tested viral markers of Hepatitis B virus, Hepatitis C virus, Human immunodeficiency virus, and syphilis, among which all donations were tested in Fujian, Guangdong and Zhejiang province and no less than 10% of donations from other areas were tested anti-HTLV-1/2 antibody. All donors should meet the basic requirements of blood donation in China. The legal age for blood donation in China is from 18 years old to 55 years old. If repeat donors pass the physical examination, they could donate until 60 years old, or even 65 years old in some regions. All the blood donors who donated samples several times were counted as one case in the study. Donors who were confirmed infection of HTLV-1/2 before or during the study would not donate blood anymore.

### Serologic testing

Blood samples from each blood donor were screened HTLV-1/2 antibody using one of three ELISA assays: Wantai HTLV-1/2 antibody ELISA kits (Wantai BioPharm, China), Murex HTLV I + II (Diasorin S.p.A., UK), and Foresight HTLV-1/2 antibody ELISA kits (Acon Biotech, China), which all detect both HTLV-1 and HTLV-2 antibody. All these three assays were based on a double-antigen sandwich method and approved by China National Medical Products Administration (NMPA). Any initial reactive samples should be retested twice by the same screening assay and those repeatedly reactive samples were sent to confirmatory lab to be further tested. During the period for confirmatory testing, the blood donors could not donated their blood until the confirmatory testing results came out.

Samples from Fujian, Guangdong (except Shenzhen), and Zhejiang province were confirmed in one of blood centers in each province (Xiamen Blood Center, Guangzhou Blood Center, and Blood Center of Zhejiang Province) using MP HTLV Blot 2.4 (MP Biomedicals, Singapore). HTLV-I viral proteins, derived from native, inactivated viral lysate and HTLV-I/II genetically engineered proteins were incorporated into the strips. Five antigens, rgp46-I, rgp46-II, GD21, p19, and p24 were used to confirm the presence of anti-HTLV-1/2 antibody.

In other areas, all screening reactive samples were further tested in the National Center for Clinical Laboratories (NCCL). Four different serologic assays were performed on one sample simultaneously. Among them two were ELISA assays: Avioq HTLV-I/II Microelisa System (abbreviated as R1, Avioq, North Carolina) and Murex HTLV I + II (R2, Diasorin S.p.A., UK). The other were based on electro-chemiluminescent or chemiluminescent method: Elecsys HTLV-I/II (R3, Roche Diagnostics, Germany) and Lumipulse G HTLV-I/II (R4, Fujirebio, Japan). Any reactive sample tested by any of the four assays above were confirmed and discriminated by Innogenetics line immunoassay (INNO-LIA HTLV I/II Score, Fujirebio, Japan). Two *gag* antigens (p19 I/II and p24 I/II) and two *env* antigens (gp46 I/II and gp21 I/II) were applied as non-type specific antigens to confirm the presence of anti-HTLV I/II. Three type-specific antigens, *gag* p19-I, *env* gp46-I, and *env* gp46-II were aimed to differentiate between HTLV-1 and HTLV-2 infections. All the assays using for supplemental tests were approved by the Food and Drug Administration (FDA) or the Conformite Europeenne (CE) and operated strictly according to the manufactures’ instructions. All the HTLV-1/2 antibody assays used in the study were summarized in Table 4.

**Table 4:** HTLV-1/2 antibody assays used in the study

Assay	Manufacturer	Assay type	Used antigens	Classification
Wantai HTLV-1/2 antibody ELISA kits	Wantai BioPharm, China	Double-antigen sandwich ELISA	Viral recombinant antigens gp21	Screening assay in blood establishments
Foresight HTLV-1/2 antibody ELISA kits	Acon Biotech, China	Double-antigen sandwich ELISA	Viral recombinant antigens gp21 and gp46	Screening assay in blood establishments
Murex HTLV I + II kits	Diasorin S.p.A., UK	Double-antigen sandwich ELISA	Synthetic *env* peptides of HTLV-1 and HTLV-2 and recombinant *gag* protein gp41 of HTLV-2	Screening assay in blood establishments; supplemental testing assay in NCCL
Avioq HTLV-I/II Microelisa System	Avioq, North Carolina, United State	Double-antigen sandwich ELISA	Viral antigens (purified viral lysate) and recombinant HTLV-1 p21E antigen	Supplemental testing assay in NCCL
Elecsys HTLV-I/II	Roche Diagnostics, Germany	Electro-chemiluminescent (ECLIA)	Viral recombinant antigens gp21 and p24	Supplemental testing assay in NCCL
Lumipulse G HTLV-I/II	Fujirebio, Japan	Chemiluminescent (CLIA)	Recombinant antigens p19 I/II, p24 I/II, and gp21 I	Supplemental testing assay in NCCL
MP HTLV Blot 2.4	MP Biomedicals, Singapore	Western Blot (WB)	Recombinant HTLV-1/2 antigens and HTLV-1 viral lysate	Confirmatory testing assay in Fujian, Guangdong and Zhejiang
INNO-LIA HTLV I/II Score	Fujirebio, Japan	Line immunoassay	Recombinant antigens p19 I/II, p24 I/II, gp46 I/II, and gp21 I/II	Confirmatory testing assay in NCCL

### Quantitative PCR

All the indeterminate donations were tested proviral DNA by an in-house real-time PCR method based on Taqman probe targeting HTLV-1 pol region [33]. Total DNA was extracted from 200 μl of whole blood of each indeterminate sample using Tiangen Magnetic Blood Genomic DNA Kit (Tiangen Biotech, China). Approximate 1 µg of total DNA was used in the real-time PCR mixture. The RPPH1 gene was amplified as an internal control. The detection limit of the qPCR method was 2.5 copies per 10,000 peripheral blood mononuclear cells.

### Follow-up of blood donors

All the information of donor’s age, sex, ethnicity, and donation history were collected from the laboratory system. Donors confirmed positive in NCCL were contacted by staff in blood establishments to complete a further targeted questionnaire. Donors confirmed indeterminate in NCCL were followed once more than 2 months after their last donation.

### Statistical analysis

Logistic regression analysis was used to investigate the role of individual demographic factors on the outcome of HTLV infections using SPSS version 21.0 (IBM Corp., Armonk, NY, USA). p < 0.05 was considered statistically significant.

## Supplementary Information

**Additional file 1: Table S1.** Repeatedly reactive donations (n=63) by confirmatory test

## Data Availability

All data generated or analyzed during this study are included in this published article [and its supplementary information files].

## Abbreviations

HTLV: Human T-lymphotropic virus
ATL: Adult T-cell leukemia lymphoma
HAM/TSP: HTLV-1 associated myelopathy or tropical spastic paraparesis
OR: Odd ratios
S/CO: Signal to cutoff value
COI: Cutoff index
NMPA: China National Medical Products Administration
NCCL: National Center for Clinical Laboratories
FDA: Food and Drug Administration
CE: Conformite Europeenne
