# Supplementary material for: Seroprevalence of human T-lymphotropic virus infection among blood donors in China: a first nationwide survey
Source: Retrovirology. 2021 Jan 7;18:2. doi: 10.1186/s12977-020-00546-w (PMC7791705; doi:10.1186/s12977-020-00546-w)
Supplement: Supplementary file 1 — Additional file 1: Table S1. Repeatedly reactive donations (n=63) by confirmatory test [file 12977_2020_546_MOESM1_ESM.docx]

Seroprevalence of human T-lymphotropic virus infection among blood donors in China: a first nationwide survey

Additional Table S1. repeatedly reactive donations (n=63) by confirmatory test

| No. | Province/City | Sex | Age | Supplemental tests | | | |  |  | INNO-LIA | | | | | | | | Result |
| --- | --- | --- | --- | --- | --- | --- | --- | --- | --- | --- | --- | --- | --- | --- | --- | --- | --- | --- |
|  |  |  |  |  |  |  |  |  | Confirmation | | | | |  | Discrimination | | |  |
|  |  |  |  | R1 | R2 | R3 | R4 |  | p19 I/II | | p24 I/II | gp46 I/II | gp21 I/II |  | p19-I | gp46-I | gp46-II |  |
| 3 | Beijing | F | 25 | R | R | R | N/A |  | ± | | ± | 1+ | － |  | － | 1+ | － | HTLV-1 |
| 4 | Beijing | F | 42 | R | R | R | R |  | 3+ | | 2+ | 3+ | 3+ |  | 2+ | 3+ | － | HTLV-1 |
| 7 | Beijing | M | 25 | R | R | R | R |  | 2+ | | 2+ | 2+ | 2+ |  | 2+ | 3+ | 1+ | HTLV-1 |
| 36 | Henan | M | 41 | R | R | R | R |  | 2+ | | 1+ | 2+ | 2+ |  | 1+ | 2+ | － | HTLV-1 |
| 88 | Jilin | M | 47 | R | R | R | R |  | 2+ | | 2+ | 3+ | 2+ |  | 2+ | 3+ | － | HTLV-1 |
| 94 | Heilongjiang | F | 35 | NR | R | R | R |  | 1+ | | － | 3+ | 2+ |  | － | 3+ | － | HTLV-1 |
| 104 | Hunan | M | 40 | R | R | R | N/A |  | 2+ | | － | ± | 2+ |  | 1+ | － | － | HTLV-1 |
| 107 | Hunan | M | 41 | R | R | R | N/A |  | － | | － | 3+ | 2+ |  | － | 3+ | － | HTLV-1 |
| 109 | Hunan | M | 52 | R | R | R | R |  | 2+ | | 2+ | 2+ | 3+ |  |  | 2+ | － | HTLV-1 |
| 146 | Guangxi | M | 44 | R | R | R | R |  | 3+ | | 3+ | 3+ | 3+ |  | 3+ | 3+ | － | HTLV-1 |
| 148 | Guangxi | F | 48 | R | R | R | R |  | 3+ | | 2+ | 2+ | 2+ |  | 2+ | 3+ | － | HTLV-1 |
| 153 | Guangxi | M | 26 | R | R | R | R |  | 3+ | | 2+ | 3+ | 3+ |  | 2+ | 3+ | － | HTLV-1 |
| 163 | Jiangsu | F | 40 | R | R | R | R |  | 3+ | | 2+ | 3+ | 3+ |  | 2+ | 3+ | － | HTLV-1 |
| 186 | Jiangxi | M | 48 | NR | R | R | R |  | 2+ | | － | 2+ | 3+ |  | － | 2+ | － | HTLV-1 |
| 188 | Jiangxi | M | 40 | R | R | R | R |  | 2+ | | 2+ | 2+ | 3+ |  | 2+ | 3+ | － | HTLV-1 |
| 189 | Jiangxi | F | 41 | R | R | R | R |  | 2+ | | 2+ | 3+ | 3+ |  | 2+ | 3+ | － | HTLV-1 |
| 220 | Hebei | F | 43 | R | R | R | R |  | 2+ | | － | 2+ | 2+ |  | － | 1+ | － | HTLV-1 |
| 383 | Hebei | M | 45 | NR | R | R | N/A |  | 3+ | | 2+ | 3+ | 3+ |  | 2+ | 3+ | － | HTLV-1 |
| 409 | Guizhou | F | 42 | R | R | R | R |  | 2+ | | 1+ | 1+ | 2+ |  | 2+ | 1+ | － | HTLV-1 |
| 434 | Xinjiang | F | 46 | R | R | R | R |  | 3+ | | 2+ | 3+ | 3+ |  | 3+ | 3+ | － | HTLV-1 |
| 525 | Sichuan | F | 40 | NR | NR | R | R |  | － | | － | 2+ | ± |  | － | 2+ | － | HTLV-1 |
| 539 | Hunan | F | 47 | R | R | NR | N/A |  | 2+ | | 1+ | 3+ | 3+ |  | 2+ | 3+ | 2+ | HTLV-1 |
| 540 | Hunan | M | 53 | R | R | N/A | R |  | 2+ | | 2+ | 3+ | 3+ |  | 2+ | 3+ | ± | HTLV-1 |
| 595 | Guangdong | M | 25 | R | R | R | N/A |  | 3+ | | 1+ | 3+ | 1+ |  | 2+ | 2+ | － | HTLV-1 |
| 600 | Guangdong | M | 27 | R | R | R | N/A |  | 2+ | | 1+ | 2+ | 2+ |  | 2+ | 3+ | － | HTLV-1 |
| 605 | Guangdong | F | 29 | R | R | R | R |  | 2+ | | － | 2+ | 2+ |  | － | 2+ | － | HTLV-1 |
| 649 | Shaanxi | F | 29 | R | R | R | R |  | 2+ | | － | 2+ | 2+ |  | ± | 2+ | － | HTLV-1 |
| 756 | Beijing | M | 35 | R | R | R | N/A |  | 2+ | | － | 2+ | 2+ |  | 2+ | 1+ | － | HTLV-1 |
| 760 | Gansu | M | 21 | NR | R | R | N/A |  | 2+ | | 2+ | 1+ | 2+ |  | 2+ | 1+ | － | HTLV-1 |
| 907 | Guangdong | M | 26 | R | R | N/A | R |  | 2+ | | 1+ | 2+ | 2+ |  | － | 2+ | － | HTLV-1 |
| 918 | Guangdong | N/A | N/A | R | R | R | R |  | 3+ | | 1+ | 2+ | 3+ |  | 2+ | 3+ | － | HTLV-1 |
| 919 | Guangdong | N/A | N/A | R | R | N/A | N/A |  | 4+ | | 2+ |  | 1+ |  | 3+ | － | － | HTLV-1 |
| 956 | Jilin | M | 54 | R | R | R | R |  | 2+ | | 2+ | 2+ | 2+ |  | － | 2+ | － | HTLV-1 |
| 960 | Jilin | F | 42 | R | R | R | R |  | 3+ | | 2+ | 2+ | 3+ |  | 3+ | 3+ | － | HTLV-1 |
| 1081 | Hunan | M | 26 | R | R | R | R |  | 2+ | | 2+ | 2+ | 3+ |  | 2+ | 3+ | － | HTLV-1 |
| 1082 | Hunan | M | 48 | NR | NR | R | R |  | 2+ | | 1+ | 2+ | 3+ |  | 1+ | 2+ | － | HTLV-1 |
| 1113 | Jiangxi | M | 46 | R | R | R | R |  | 3+ | | 2+ | 3+ | 3+ |  | 2+ | 3+ | － | HTLV-1 |
| 1120 | Guangxi | F | 49 | R | R | R | R |  | 3+ | | 2+ | 3+ | 3+ |  | 2+ | 3+ | － | HTLV-1 |
| 1124 | Guizhou | F | 38 | R | R | R | R |  | 3+ | | 2+ | 3+ | 3+ |  | 2+ | 3+ | － | HTLV-1 |
| 1169 | Jiangsu | F | 26 | R | R | R | R |  | 3+ | | 2+ | 3+ | 3+ |  | 2+ | 3+ | － | HTLV-1 |
| 1197 | Guangdong | F | 30 | R | R | R | R |  | 2+ | | － | 2+ | 3+ |  | ± | － | － | HTLV-1 |
| 1198 | Guangdong | M | 26 | R | R | R | R |  | 2+ | | 2+ | 2+ | 3+ |  | 3+ | 2+ | － | HTLV-1 |
| 1312 | Sichuan | M | 47 | R | R | R | R |  | 3+ | | 2+ | 3+ | 3+ |  | 1+ | 3+ | － | HTLV-1 |
| 1381 | Guizhou | F | 38 | R | R | R | R |  | 2+ | | 2+ | 3+ | 3+ |  | 2+ | 3+ | － | HTLV-1 |
| 1387 | Jiangxi | M | 45 | R | R | R | R |  | 3+ | | 3+ | 2+ | 3+ |  | 2+ | 2+ | － | HTLV-1 |
| 1405 | Guangdong | N/A | N/A | R | R | R | R |  | 2＋ | | 1+ | 2+ | 2+ |  | － | 2+ | － | HTLV-1 |
| 1407 | Guangdong | N/A | N/A | R | R | R | R |  | 2＋ | | 3＋ | 3+ | 3+ |  | 2+ | 3+ | － | HTLV-1 |
| 1409 | Guangdong | N/A | N/A | R | R | R | R |  | 3+ | | 3+ | 3+ | 3+ |  | 2+ | 3+ | － | HTLV-1 |
| 1648 | Heilongjiang | F | 47 | R | R | R | R |  | 2+ | | － | 2+ | 3+ |  | 2+ | 2+ | － | HTLV-1 |
| 53 | Henan | M | 42 | NR | R | NR | R |  | － | | 1+ | － | ± |  | － | － | － | HTLV-1/2 |
| 359 | Hunan | M | 20 | R | R | R | R |  | 2+ | | 2+ | 1+ | 2+ |  | － | － | － | HTLV-1/2 |
| 457 | Jiangxi | M | 19 | NR | NR | NR | R |  | ± | | － | － | 1+ |  | － | － | － | HTLV-1/2 |
| 489 | Jilin | M | 30 | NR | NR | R | NR |  | ± | | － | － | ± |  | － | － | － | HTLV-1/2 |
| 495 | Yunan | F | 30 | NR | NR | R | R |  | 1+ | | － | － | 2+ |  | － | － | － | HTLV-1/2 |
| 503 | Hebei | M | 34 | NR | NR | R | N/A |  | － | | 1+ | － | 1+ |  | － | － | － | HTLV-1/2 |
| 647 | Heilongjiang | F | 30 | NR | NR | R | NR |  | 1+ | | － | － | ± |  | － | － | － | HTLV-1/2 |
| 816 | Guizhou | M | 47 | NR | R | NR | NR |  | 1+ | | － | － | 1+ |  | － | － | － | HTLV-1/2 |
| 1132 | Guangdong | N/A | N/A | R | R | R | R |  | 2+ | | 2+ | 1+ | 2+ |  | － | － | － | HTLV-1/2 |
| 1285 | Guangxi | M | 48 | NR | NR | R | NR |  | － | | 1+ | － | 1+ |  | － | － | － | HTLV-1/2 |
| 1295 | Sichuan | M | 53 | NR | R | NR | NR |  | ± | | ± | － | 2+ |  | － | － | － | HTLV-1/2 |
| 1412 | Jiangxi | M | 29 | NR | R | NR | R |  | 2+ | | － | － | 1+ |  | － | － | － | HTLV-1/2 |
| 1441 | Jiangsu | F | 43 | NR | NR | R | R |  | 2+ | | － | － | 1+ |  | － | － | － | HTLV-1/2 |
| 1461 | Hebei | F | 21 | NR | NR | R | NR |  | － | | 2+ | － | ± |  | － | － | － | HTLV-1/2 |

R: reactive, S/CO or COI >=1.0; NR: nonreactive, S/CO or COI< 1.0. N/A: Not available. F, Female; M, Male.

Supplemental testing assays: R1, Avioq HTLV-I/II Microelisa System (Avioq, North Carolina, United State); R2, Murex HTLV I+II (Diasorin S.p.A., UK); R3,

Elecsys HTLV-I/II (Roche Diagnostics, Germany); R4, Lumipulse G HTLV-I/II (Fujirebio, Japan)
